# Effect of relaxin‐3 on Kiss‐1, gonadotropin‐releasing hormone, and gonadotropin subunit gene expression

**DOI:** 10.1002/rmb2.12298

**Published:** 2019-09-05

**Authors:** Haruhiko Kanasaki, Tuvshintugs Tumurbaatar, Zolzaya Tumurgan, Aki Oride, Hiroe Okada, Satoru Kyo

**Affiliations:** ^1^ Department of Obstetrics and Gynecology Shimane University School of Medicine Izumo Japan

**Keywords:** gonadotropin, gonadotropin‐releasing hormone, hypothalamic‐pituitary‐gonadal axis, kisspeptin, relaxin‐3

## Abstract

**Purpose:**

Relaxin‐3 is a hypothalamic neuropeptide that belongs to the insulin superfamily. We examined whether relaxin‐3 could affect hypothalamic Kiss‐1, gonadotropin‐releasing hormone (GnRH), and pituitary gonadotropin subunit gene expression.

**Methods:**

Mouse hypothalamic cell models, mHypoA‐50 (originated from the hypothalamic anteroventral periventricular region), mHypoA‐55 (originated from arcuate nucleus), and GT1‐7, and the mouse pituitary gonadotroph LβT2 were used. Expression of Kiss‐1, GnRH, and luteinizing hormone (LH)/follicle‐stimulating hormone (FSH) β‐subunits was determined after stimulation with relaxin‐3.

**Results:**

RXFP3, a principle relaxin‐3 receptor, was expressed in these cell models. In mHypoA‐50 cells, relaxin‐3 did not exert a significant effect on Kiss‐1 expression. In contrast, the Kiss‐1 gene in mHypoA‐55 was significantly increased by 1 nmol/L relaxin‐3. These cells also express GnRH mRNA, and its expression was significantly stimulated by relaxin‐3. In GT1‐7 cells, relaxin‐3 significantly upregulated Kiss‐1 expression; however, GnRH mRNA expression in GT1‐7 cells was not altered. In primary cultures of fetal rat neuronal cells, 100 nmol/L relaxin‐3 significantly increased GnRH expression. In pituitary gonadotroph LβT2, both LHβ‐ and FSHβ‐subunit were significantly increased by 1 nmol/L relaxin‐3.

**Conclusions:**

Our findings suggest that relaxin‐3 exerts its effect by modulating the expression of Kiss‐1, GnRH, and gonadotropin subunits, all of which are part of the hypothalamic‐pituitary‐gonadal axis.

## 1 INTRODUCTION

It is well established that female reproduction is under the dynamic control of hypothalamic gonadotropin‐releasing hormone (GnRH) and pituitary gonadotropin secretion via regulatory feedback loops mediated by sex steroid hormones within the hypothalamic‐pituitary‐gonadal (HPG) axis. Hypothalamic kisspeptin, which is encoded by the Kiss‐1 gene, is currently believed to be positioned at the highest level in the HPG axis and controls GnRH.^1^ GnRH released into portal circulation, in turn, reaches the anterior pituitary and stimulates the synthesis and release of the gonadotropins luteinizing hormone (LH) and follicle‐stimulating hormone (FSH). Luteinizing hormone and FSH individually or cooperatively act on gonads to regulate gametogenesis and steroidogenesis. In mammals, Kiss‐1 neurons, which regulate GnRH, are located in two different areas of the hypothalamus, the anteroventral periventricular (AVPV) and arcuate nucleus (ARC).^2^ At present, it is speculated that Kiss‐1 neurons in the AVPV region are responsible for the estradiol‐induced (E2‐induced) GnRH‐LH surge (positive feedback), whereas those in the ARC region are involved in an E2‐induced negative feedback mechanism. This concept is based on the observations that Kiss‐1 expression in the AVPV region is upregulated by E2, whereas that in the ARC is inhibited by E2.^3^

Relaxin‐3 is a hypothalamic neuropeptide that belongs to the insulin superfamily.^4^ Prior to its discovery, it was known that there are two genes encoding relaxin‐1 and ‐2.^5^, ^6^ Unlike relaxin‐1 and ‐2, relaxin‐3 is predominantly expressed in the brain, with particularly strong expression in the pontine nucleus incertus (NI).^4^ Classically, relaxin‐1 and ‐2 have been known to be produced by the ovary and target the mammalian reproductive system to ripen the uterine cervix, elongate the pubic symphysis, and inhibit uterine contraction. They also have roles in enhancing sperm motility and regulating blood pressure.^7^ As for the characteristics of relaxin‐3, it seems to have orexigenic effects; however, the details of the physiological role of relaxin‐3 are largely unknown. Previous literature reported that chronic intracerebroventricular injection of relaxin‐3 induced hyperphagia and increased body weight and fat mass in male rats.^8^ Repeated relaxin‐3 administration has also been reported to increase cumulative food intake with an increase in leptin serum level in male rats.^9^ Chronic stress and repeated food restriction have been reported to increase body weight in female rats, which was associated with a significant increase in relaxin‐3 mRNA levels in the brain.^10^ Relaxin‐3 is expressed in the NI of the brain stem, which has projections to many hypothalamic areas, and it has also been found via immunostaining in the paraventricular nucleus (PVN), ARC, and preoptic area (POA), all of which are areas known to be important in the regulation of the HPG axis.^11^

In 2008, McGowan et al demonstrated that intracerebroventricular administration of relaxin‐3 to adult male rats significantly increased plasma levels of LH. Because this effect was blocked by pretreatment with a peripheral GnRH agonist, they concluded that relaxin‐3 stimulates the reproductive axis by stimulating GnRH neurons. Using hypothalamic explants and a mouse GnRH‐producing hypothalamic cell model, namely GT1‐7 cells, they demonstrated that relaxin‐3 stimulates the release of GnRH.^12^

Relaxin‐3 binds mainly to the receptor RXFP3,^13^ but it also binds and activates the receptor RXFP1.^14^ These receptors are expressed predominantly in the central nervous system, particularly in the PVN, POA, and ARC regions of the hypothalamus.^15^, ^16^, ^17^ These areas are known to play an important role in the regulation of appetite and/or the HPG axis. Although relaxin‐3 binds to both RXFP3 and RXFP1, RXFP1 does not participate in appetite control in rats.^8^ RXFP3 exerts its receptor effects by inhibiting cAMP, normally associated with Gi‐protein coupling,^15^ whereas RXFP1 is thought to mediate Gs‐protein‐dependent modulation of cAMP production.^18^ In the present study, we focused on the functional role of relaxin‐3/RXFP3 in the components of the HPG axis by investigating the direct effect of relaxin‐3 on Kiss‐1, GnRH, and pituitary gonadotropin subunit expression using hypothalamic neuronal cell models and a pituitary gonadotroph cell model.

## 2 MATERIALS AND METHODS

### 2.1 Materials and cell models

The following chemicals and reagents were obtained from the indicated sources: Gibco fetal bovine serum (FBS) (Invitrogen, Carlsbad, CA); relaxin‐3 (Phoenix Pharmaceuticals, Inc); and penicillin‐streptomycin (Sigma‐Aldrich Co.). mHypoA‐50 and mHypoA‐55 cells were purchased from Cedarlane Corp. GT1‐7 and LβT2 cells were kindly provided by Dr P. Mellon of the University of California, San Diego.

### 2.2 Primary culture of fetal rat brain cells

Fetal rat brains (n = 6‐8) were obtained from the fetuses of female rats at 16‐18 days of gestation under deep sodium pentobarbital anesthesia. Whole brains were excised and minced before incubating in Ca^2+^‐ and Mg^2+^‐free Hank's balanced salt solution (CMF‐HBSS) containing 10 mg/mL trypsin and 2 mg/mL collagenase (Nitta Gelatin) for 15 minutes at 37°C. Samples were then incubated in an identical solution containing 0.5 μg/mL DNase I (Boehringer‐Mannheim) for 5 minutes at 37°C. After incubation in CMF‐HBSS containing 5 mmol/L ethylenediaminetetraacetic acid (Wako Pure Chemicals) for 5 minutes at 37°C, the samples were washed with CMF‐HBSS. Dispersed cells were then suspended in CMF‐HBSS using a pipette, passed through a 70‐μm nylon mesh (Becton Dickinson Labware), and then collected by centrifugation. The pellet was suspended, and 2‐3 × 10^5^ cells were cultured on a 35‐mm petri dish in DMEM with 10% FBS and 1% penicillin‐streptomycin until use. This protocol was approved by the committee of the Experimental Animal Center for Integrated Research at Shimane University.

### 2.3 Cell culture and stimulation

Cells were plated in 35‐mm tissue culture dishes and incubated with high‐glucose DMEM containing 10% heat‐inactivated FBS and 1% penicillin‐streptomycin at 37°C under a humidified atmosphere of 5% CO_2_ in air. After 24 hours, cells were used for each experiment. While stimulated with the test reagents, cells were incubated with or without (control) the test reagents in high‐glucose DMEM containing 1% heat‐inactivated FBS and 1% penicillin‐streptomycin for the indicated concentrations and time periods.

### 2.4 Western blot analysis

Cell extracts were lysed on ice with RIPA buffer (phosphate‐buffered saline, 1% NP‐40, 0.5% sodium deoxycholate, and 0.1% sodium dodecyl sulfate [SDS]) containing 0.1 mg/mL phenylmethyl sulfonyl fluoride, 30 mg/mL aprotinin, and 1 mmol/L sodium orthovanadate, scraped for 20 seconds, and centrifuged at 14 000 *g* for 10 minutes at 4°C. Protein concentration in the cell lysates was measured using the Bradford method. Denatured protein (30 µg per well) was resolved in 10% SDS polyacrylamide gel electrophoresis (SDS‐PAGE) gels according to standard protocols. Protein was then transferred onto polyvinylidene difluoride membranes (Hybond‐P PVDF; Amersham Biosciences), which were blocked for 2 hours at room temperature in Blotto (5% milk in Tris‐buffered saline). Membranes were incubated with anti‐RXFP3 antibody (1:100 dilution; Santa Cruz Biotechnology, Inc) in Blotto overnight at 4°C and washed three times for 10 minutes per wash with Tris‐buffered saline/1% Tween. Subsequent incubation with horseradish peroxidase‐conjugated (HRP‐conjugated) antibodies was performed for 1 hour at room temperature in Blotto, and additional washes were performed as needed. Following enhanced chemiluminescence detection (Amersham Biosciences), membranes were exposed to X‐ray film (Fujifilm). Tissues from rat cerebral cortex were used as positive controls.

### 2.5 RNA preparation, reverse transcription, PCR, and quantitative real‐time PCR

Total RNA from stimulated cells was extracted using TRIzol‐LS (Invitrogen) according to the manufacturer's instructions. To obtain cDNA, 1.0 µg of total RNA was reverse transcribed in RT buffer using an oligo‐dT primer (Promega, Madison, WI) and a First‐Strand cDNA Synthesis Kit (Invitrogen). The preparation was supplemented with 10 mmol/L dithiothreitol, 1 mmol/L of each dNTP, and 200 U RNase inhibitor/human placenta ribonuclease inhibitor (Code No. 2310; Takara) in a final volume of 10 µL. The reaction was incubated at 37°C for 60 minutes. For the detection of Rxfp3 mRNA, after PCR amplification using primers for Rxfp3 (forward: 5′‐TGCTGGGCCTCCTGCTGCTGCTGAGCATCATCA‐3′ and reverse: CTTGCGGAACTCGCGGCGCACTAAGCAGTAGAGGAT‐3′), amplicons were electrophoresed in agarose gels and visualized with ethidium bromide staining. cDNAs from rat cerebral cortex and from COS7 cells were used as positive and negative controls, respectively. Quantification of Kiss‐1, GnRH, LHβ‐, and FSHβ‐subunit mRNAs was obtained through quantitative real‐time PCR (ABI Prism 7000; Perkin‐Elmer Applied Biosystems) following the manufacturer's protocol (User Bulletin No. 2) and utilizing Universal ProbeLibrary Probes and FastStart Master Mix (Roche Diagnostics). Using specific primers for mouse Kiss‐1 (forward: 5′‐ATGATCTCGCTGGCTTCTTGG‐3′; reverse: 5′‐GGTTCACCACAGGTGCCATTTT‐3′), GnRH (forward: 5′‐ACTGTGTGTTTGGAAGGCTGC‐3′ and reverse: 5′‐TTCCAGAGCTCCTCGCAGATC‐3′), LHβ (forward: 5′‐GCCGGCCTGTCAACGCAACC‐3′; reverse: 5′‐GAGGGCCACAGGGAAGGAGA‐3′), and FSHβ (forward: 5′‐ACCATGATGAAGTTGATCCAG‐3′; reverse: 5′‐TCCTTCATTTCACTGAAGGAG‐3′), the simultaneous measurement of mRNA and GAPDH permitted normalization of the amount of cDNA added per sample. For each set of primers, a no‐template control was included. Thermal cycling conditions were as follows: 10 minutes denaturation at 95°C, followed by 40 cycles of 95°C for 15 seconds and 60°C for 1 minute. Reactions were followed by melting curve analysis (55–95°C). To determine PCR efficiency, a 10‐fold serial dilution of cDNA was performed as previously described.^19^ PCR conditions were optimized to generate >95% PCR efficiency, and only those reactions with between 95% and 105% efficiency were included in subsequent analyses. Relative differences in cDNA concentration between baseline and experimental conditions were then calculated using the comparative threshold cycle (Ct) method.^20^ Briefly, for each sample, a ΔCt was calculated to normalize to the internal control using the following equation: ΔCt = ΔCt(gene) – Ct (GAPDH). To obtain differences between experimental and control conditions, ΔΔCt was calculated as ΔCt(sample) – ΔCt(control). Relative mRNA levels were then calculated using the following equation: fold difference = 2^ΔΔCt^.

### 2.6 Statistical analysis

All experiments were repeated independently at least three times. Each experiment was performed using duplicate samples in each experimental group. When we determined the mRNA expression, two samples were assayed in duplicate. Six averages from three independent experiments were statistically analyzed. Data are expressed as the mean ± standard error of the mean (SEM) values. Statistical analysis was performed using one‐way analysis of variance (ANOVA) with Bonferroni's post hoc test. *P* < .05 was considered statistically significant.

## 3 RESULTS

### 3.1 Expression of relaxin‐3 receptor RXFP3 in hypothalamic and pituitary gonadotroph cell models

**Figure 1:**
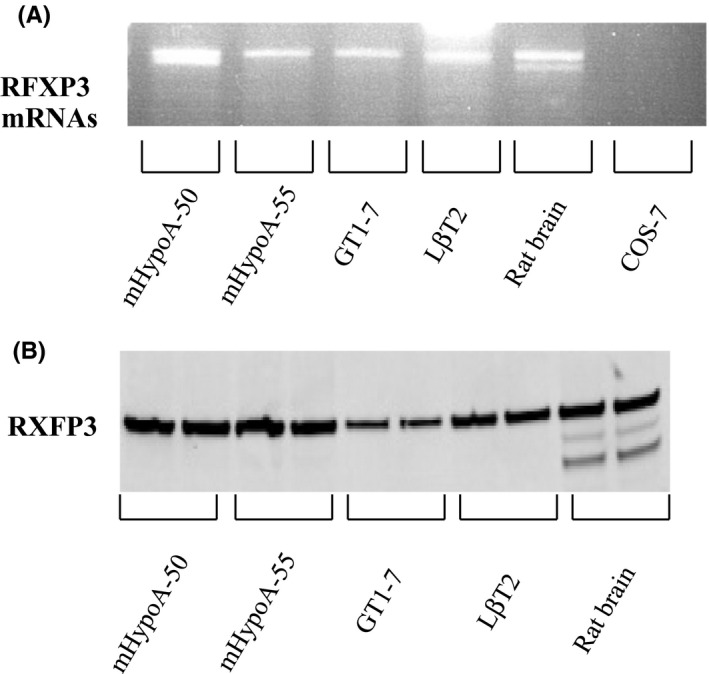
Expression of the relaxin‐3 receptor RXFP3 in the hypothalamic and pituitary gonadotroph cell models. A, Total RNA was prepared, and RT‐PCR was carried out for 40 cycles using Rcfp3‐specific primers. PCR products were resolved in a 1.5% agarose gel and visualized with ethidium bromide staining. cDNAs from COS7 cells were used as the negative control. B, Cell lysates (30 μg protein) from the model cells were analyzed by SDS‐PAGE followed by immunoblotting and incubation with an antibody against RXFP3. The bands were visualized using an HRP‐conjugated secondary antibody

As neuronal cell models from the hypothalamus, we used mHypoA‐50 and mHypoA‐55 cells. These cells originate from the AVPV and ARC regions of the hypothalamus, and respond differently to E2.^21^ We also used GT1‐7 hypothalamic neurons and the pituitary gonadotroph cell model LβT2. RT‐PCR using specific primers for the relaxin‐3 receptor RXFP3 demonstrated that all of these cells express the RXFP3 gene (Figure 1A). Western blotting analysis using anti‐RXFP3 antibody revealed that RXFP3 protein was also expressed in these cell models (Figure 1B). Extracts or cDNAs from rat brain tissue were used as positive controls.

### 3.2 Effect of relaxin‐3 on Kiss‐1 gene expression in mHypoA‐50 and mHypoA‐55 cells

**Figure 2:**
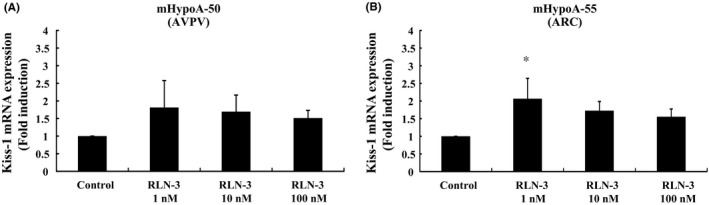
Effect of relaxin‐3 on Kiss‐1 mRNA expression in mHypoA‐50 and mHypoA‐55 cells. mHypoA‐50 AVPV cells (A) and mHypoA‐55 ARC cells (B) were stimulated with the indicated concentrations of relaxin‐3 (RLN‐3) for 24 h. mRNA was then extracted and reverse transcribed, and Kiss‐1 mRNA levels were measured by quantitative real‐time PCR. Results are expressed as the fold induction over unstimulated cells and presented as mean ± SEM values of three independent experiments, each performed with duplicate samples. *P < .05 vs control. Statistical significance was determined by one‐way ANOVA with Bonferroni's post hoc test

To examine whether relaxin‐3 could modify the expression of Kiss‐1, we stimulated the mHypoA‐50 and mHypoA‐55 cells with relaxin‐3 for 24 hours. In mHypoA‐50 AVPV model cells, relaxin‐3 did not exert a significant effect on Kiss‐1 expression (Figure 2A). In contrast, in mHypoA‐55 ARC model cells, 1 nmol/L relaxin‐3 significantly increased Kiss‐1 gene expression 2.07 ± 0.58‐fold (Figure 2B).

### 3.3 Effect of relaxin‐3 on GnRH gene expression in mHypoA‐50 and mHypoA‐55 cells

**Figure 3:**
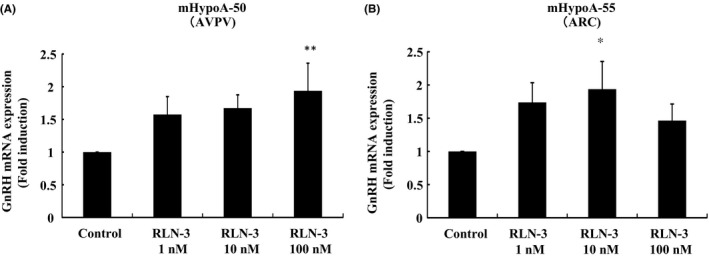
Effect of relaxin‐3 on GnRH mRNA expression in mHypoA‐50 and mHypoA‐55 cells. mHypoA‐50 AVPV cells (A) and mHypoA‐55 ARC cells (B) were stimulated with the indicated concentrations of relaxin‐3 (RLN‐3) for 24 h. mRNA was then extracted and reverse transcribed, and GnRH mRNA levels were measured by quantitative real‐time PCR. Results are expressed as the fold induction over unstimulated cells and presented as mean ± SEM values of three independent experiments, each performed with duplicate samples. **P < .01, *P < .05 vs control. Statistical significance was determined by one‐way ANOVA with Bonferroni's post hoc test

Both mHypoA‐50 ARC and mHypoA‐55 AVPV cells also express GnRH. Our results show that relaxin‐3 has an effect on GnRH expression in these cells. Relaxin‐3 at 100 nmol/L significantly increased GnRH mRNA expression 1.94 ± 0.42‐fold in mHypoA‐50 AVPV cells (Figure 3A). Similarly, GnRH mRNA expression in mHypoA‐55 ARC cells was significantly increased 1.94 ± 0.42‐fold by 10 nmol/L relaxin‐3 (Figure 3B).

### 3.4 Effect of relaxin‐3 on Kiss‐1 and GnRH gene expression in GT1‐7 hypothalamic model cells

**Figure 4:**
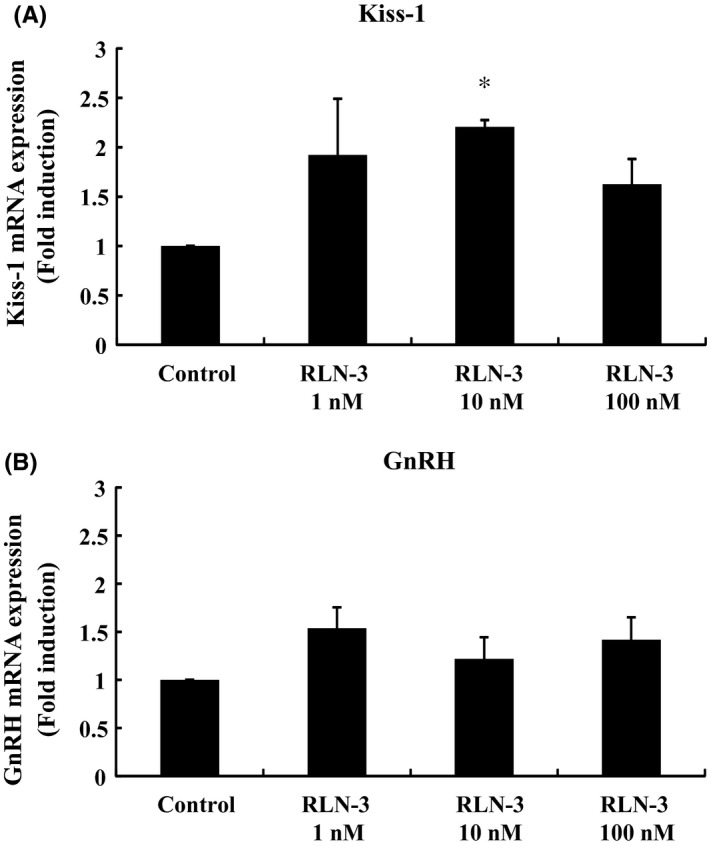
Effect of relaxin‐3 on Kiss‐1 and GnRH mRNA expression in GT1‐7 cells. GT1‐7 cells were stimulated with the indicated concentrations of relaxin‐3 (RLN‐3) for 24 h. mRNA was then extracted and reverse transcribed, and Kiss‐1 (A) and GnRH (B) mRNA levels were measured by quantitative real‐time PCR. Results are expressed as the fold induction over unstimulated cells and presented as mean ± SEM values of three independent experiments, each performed with duplicate samples. *P < .05 vs control. Statistical significance was determined by one‐way ANOVA with Bonferroni's post hoc test

GT1‐7 cells were created from a transgenic mouse utilizing 5′‐flanking DNA of the rat GnRH gene to target expression of SV40 T‐antigen in GnRH neurons.^22^ Although GT1‐7 cells have been widely used as a GnRH‐producing cell model, they also express the Kiss‐1 gene.^23^ Similar to the phenomenon observed in mHypo‐A55 cells, 10 nmol/L relaxin‐3 treatment resulted in a 2.21 ± 0.07‐fold increase in Kiss‐1 gene expression in GT1‐7 cells (Figure 4A). However, in GT1‐7 cells, relaxin‐3 failed to increase GnRH mRNA expression (Figure 4B).

### 3.5 Effect of relaxin‐3 on GnRH gene expression in primary cultures of fetal rat neuronal cells

**Figure 5:**
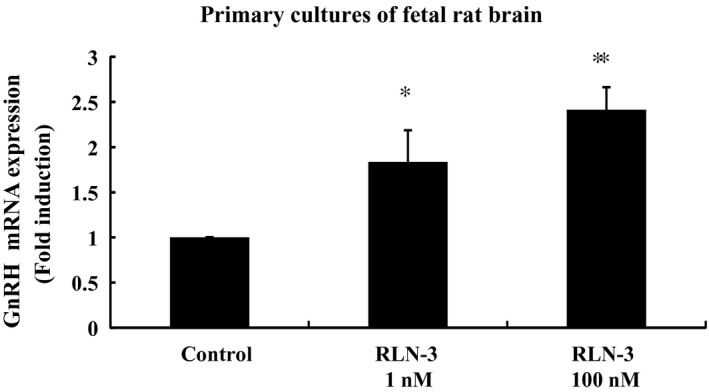
Effect of relaxin‐3 on GnRH mRNA expression in primary cultures of fetal rat brain. Neuronal cells from fetal rat brain were stimulated with the indicated concentrations of relaxin‐3 (RLN‐3) for 24 h. mRNA was then extracted and reverse transcribed, and GnRH mRNA levels were measured by quantitative real‐time PCR. Results are expressed as the fold induction over unstimulated cells and presented as mean ± SEM values of three independent experiments, each performed with duplicate samples. **P < .01, *P < .05 vs control. Statistical significance was determined by one‐way ANOVA with Bonferroni's post hoc test

To further examine the effect of relaxin‐3 on GnRH mRNA expression, we used primary cultures of neuronal cells from fetal rat brain. These cells contain GnRH‐expressing neurons. Similar to our observations in mHypoA‐50 and mHypoA‐55 cells, relaxin‐3 treatment increased GnRH mRNA expression in fetal rat neuronal cells 1.84 ± 0.35‐fold at 1 nmol/L and 2.41 ± 0.35‐fold at 100 nmol/L (Figure 5).

### 3.6 **Effect of relaxin‐3 on the expression of gonadotropin LH**β**‐ and FSH**β**‐subunits**

**Figure 6:**
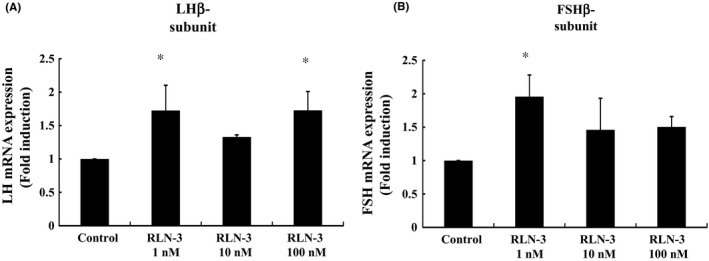
Effect of relaxin‐3 on gonadotropin subunit mRNA expression in LβT2 cells. LβT2 cells were stimulated with the indicated concentrations of relaxin‐3 (RLN‐3) for 24 h. mRNA was then extracted and reverse transcribed, and GnRH mRNA levels were measured by quantitative real‐time PCR. Results are expressed as the fold induction over unstimulated cells and presented as mean ± SEM values of three independent experiments, each performed with duplicate samples. *P < .05 vs control. Statistical significance was determined by one‐way ANOVA with Bonferroni's post hoc test

Lastly, we examined the effect of relaxin‐3 on the expression of pituitary gonadotropin LHβ‐ and FSHβ‐subunits using a gonadotroph cell model. These subunits determine the specificity of LH and FSH by binding the α‐subunit, which is common to both LH and FSH. Our results show that relaxin‐3 also has an effect on gonadotropin subunit gene expression. In the mouse gonadotroph cell model LβT2, 1 nmol/L relaxin‐3 increased LHβ‐subunit mRNA expression 1.73 ± 0.34‐fold, which was statistically significant (Figure 6A). Similarly, 1 nmol/L relaxin‐3 significantly increased FSHβ‐subunit expression by 1.96 ± 0.32‐fold in these gonadotrophs (Figure 6B).

## 4 DISCUSSION

Energy balance and reproduction are intrinsically linked, and a number of food‐intake–related hormones such as leptin and ghrelin have been reported to be involved in controlling the reproductive axis.^24^ Neuropeptide Y and galanin‐like peptide can also modulate the HPG axis.^25^, ^26^ McGowan et al^8^, ^9^ first reported that central administration of relaxin‐3 increases food intake. They also reported that (a) central injection of relaxin‐3 increases serum levels of LH, and this effect is blocked by administration of a peripheral GnRH agonist; (b) relaxin‐3 stimulates release of GnRH from hypothalamic explants and GT1‐7 cells; and (c) relaxin‐3 does not stimulate gonadotropin release from pituitary fragments.^12^ From these observations, they concluded that relaxin‐3 stimulates the HPG axis via hypothalamic GnRH neurons.

In this study, we further examined the role of relaxin‐3 using hormone‐secreting cells from the hypothalamus and pituitary because hypothalamic kisspeptin, GnRH, and pituitary gonadotrophs play pivotal roles in controlling the HPG axis. mHypoA‐50 and mHypoA‐55 are cell models that originated from the AVPV and ARC regions of the mouse hypothalamus. Although Kiss‐1 is expressed by both of these cells, mHypoA‐50 and mHypoA‐55 have distinct characters. mHypoA‐55 ARC cells express substance P, neurokinin B, and dynorphin, all of which are expressed in kisspeptin neurons in the ARC region (KNDy neurons). In these cells, the expression of Kiss‐1 is repressed by E2 under certain conditions.^21^ By contrast, mHypoA‐50 AVPV cells express tyrosine hydroxylase, but not neurokinin B and dynorphin. Also, the Kiss‐1 gene in mHypoA‐50 cells is upregulated by E2.^21^ Interestingly, although both mHypoA‐50 and mHypoA‐55 were first developed as Kiss‐1‐producing neurons, these cells also express GnRH.^27^ Similarly, another hypothalamic cell model, GT1‐7, was found to not only express GnRH, but also to express and secrete Kiss‐1/kisspeptin.^23^, ^28^ Using these cell models, we found that relaxin‐3 could be a potent stimulator of Kiss‐1 gene expression in the hypothalamus because it increased Kiss‐1 expression in mHypoA‐55 and GT1‐7 cells. At present, it is still unclear why relaxin‐3 stimulated Kiss‐1 gene expression in mHypoA‐55 ARC cells, but not in mHypoA‐50 AVPV cells. Also, it is likely that relaxin‐3 could stimulate hypothalamic GnRH expression in vivo because relaxin‐3 increased GnRH expression in mHypoA‐50 and mHypoA‐55 cells. The GnRH‐stimulating action of relaxin‐3 was also confirmed using primary cultures of fetal rat brain that possess GnRH‐producing neurons. In this study, we did not examine the changes in Kiss‐1 gene expression in primary cultures of fetal rat brain because we could not distinguish which areas of Kiss‐1 neurons respond to relaxin‐3.

The 2005 study by McGowan et al^12^ concluded that relaxin‐3 stimulates the HPG axis via GnRH neurons. This conclusion was partially supported by the observation that GT1‐7 cells responded to relaxin‐3 and stimulated the release of GnRH. Curiously, our GT1‐7 cells did not respond to relaxin‐3. It is plausible that relaxin‐3 is involved in the release of GnRH, but not in its synthesis. However, considering our previous observation that our line of GT1‐7 cells did not stimulate GnRH expression even when G protein‐coupled receptor 54 was overexpressed,^29^ our result might largely depend on the character of this cell model. Nevertheless, our experiments using fetal brain cultures might support the previous observation that relaxin‐3 stimulates GnRH neurons. As for the effect of relaxin‐3 on the pituitary gonadotrophs, a discrepancy exists between our results and those of McGowan et al Our observation using LβT2 cells showed that relaxin‐3 could stimulate the expression of LH and FSH β‐subunits. However, in McGowan's study, relaxin‐3 failed to stimulate the release of these hormones from pituitary fragments in vitro.^8^ We only examined the gene expression of gonadotropins, whereas McGowan's study determined the release of gonadotropins. If the mechanisms of release and synthesis are distinct, there is a possibility that the effect of relaxin‐3 differs between secretion and gene expression. Another possibility is that the pituitary fragments used in McGowan's study contained a variety of anterior pituitary cells that included cells other than gonadotrophs. Furthermore, connective tissue or local factors may disturb or counteract the effect of relaxin‐3 in pituitary fragments. However, at the very least, because we observed a stimulatory effect of this peptide in LHβ and FSHβ mRNA expression, it is plausible that relaxin‐3 could stimulate the population of gonadotrophs in the pituitary gland.

Relaxin‐3 immunoreactivity was present in the PVN, ARC, lateral hypothalamic area, and medial and lateral POAs, all of which are afferent to and contiguous with GnRH or Kiss‐1 neurons.^11^, ^30^ Receptors for relaxin‐3 (RXFP3 and RXFP1) are present in the hypothalamus, including the PVN, POA, and ARC regions where GnRH or kisspeptin neurons exist.^15^, ^16^, ^17^ Furthermore, relaxin‐3 receptors are also present in the pituitary gland.^31^ We also demonstrated that all of the Kiss‐1‐, GnRH‐, and gonadotropin‐subunit–expressing cell models used in this study express the relaxin‐3 receptor RXFP3 and responded to relaxin‐3.

There are several limitations of this study. We did not examine RXFP3 receptor blockage, for example, by using an anti‐RXFP3 antibody. The role of relaxin‐3 in mediating E2‐induced positive and negative feedback mechanisms remains unclear because we did not examine how relaxin‐3 itself is regulated by E2. Furthermore, relaxin‐3 had effects on Kiss‐1 expression in mHypoA‐55 and GT1‐7 cells, and on GnRH expression in both mHypoA‐50 AVPV and mHypoA‐55ARC cells as well as fetal brain cultures. However, the effect of relaxin‐3 on the expression of these genes was not dose dependent. A significant effect was only obtained at one or two concentrations in our dose‐response experiments. The effective dose of relaxin‐3 varied depending on the gene, despite using the same cell models. These results might be clarified by repeated experiments, or more precise determination might reveal the optimal relaxin‐3 concentration to induce target genes. Nevertheless, our current observations imply that relaxin‐3 affects both the hypothalamic and pituitary cells and plays a role in regulating the HPG axis.

It is plausible that relaxin‐3 is an orexigenic peptide because it induced hyperphagia whereas its antagonist induced hypophagia. Although one report showed that chronic stress and repeated food restriction increased body weight, which was associated with a significant increase in relaxin‐3 mRNA levels in female rat brain,^10^ no changes in the expression of relaxin‐3 mRNA or its receptors in satiated and starved animals have been reported to date. If relaxin‐3 plays a physiological role in controlling appetite, one might expect increased relaxin‐3 expression during food restriction and decreased in the satiated situation. However, the pulsatility of GnRH/gonadotropin secretion is known to be suppressed by starvation.^32^ Because the physiology of this peptide is still largely obscure, it would be interesting to study this peptide in starved animals. A recently published clinical study assessing serum levels of relaxin‐3 in patients with delayed puberty revealed that relaxin‐3 levels were significantly positively correlated with LH, FSH, and sex steroids.^33^ Because relaxin‐3 could stimulate the expression of Kiss‐1, GnRH, and pituitary gonadotropins by itself, it may be involved in coordinating feeding or energy balance with reproduction.

## CONFLICT OF INTEREST

All authors declare that they have no conflict of interest.

## HUMAN AND ANIMAL RIGHTS

This study's protocol was approved by the committee of the Experimental Animal Center for Integrated Research at Shimane University, Izumo, Japan. All institutional and national guidelines for the care and use of laboratory animals were followed. This article does not contain any studies with human subjects performed by any of the authors.
